# Nutrition Knowledge, English Adequacy, Women's Education, and Food Insecurity Among Syrian Refugees in Florida

**DOI:** 10.7759/cureus.69084

**Published:** 2024-09-10

**Authors:** Racha Sankar, Fatma Huffman

**Affiliations:** 1 Health and Human Performance, Parker University, Dallas, USA; 2 Dietetics and Nutrition, Florida International University, Miami, USA

**Keywords:** english proficiency, food security, nutrition knowledge, refugee, syrian, women's education

## Abstract

Objectives

The objective of the study was to measure food security among Syrian refugees residing in urban and rural areas in Florida. Women's education, English proficiency, and nutrition knowledge were assessed to indicate their effects on food security in this population.

Methods

One-on-one interview questionnaires were administered to Syrian refugee households residing in Florida (n=80: n=43 in rural areas and n=37 in urban areas). The main outcomes were food insecurity, nutrition knowledge, English adequacy, and women's education.

Results

The main outcome of this study was the food security status among Syrian refugees residing in Florida. Among the total households, 80% were food insecure, and food insecurity was greater in rural areas (60.9%) than in urban areas (39.1%). The majority (57.5%) of participants scored fair nutrition knowledge. One-way frequency analysis showed that 75% of households had inadequate English. Around 23.7% of Syrian refugee women had a high school diploma or higher. Among these women, 68.4% were residing in urban areas. The chi-squared test showed significant differences in women's education in rural and urban areas (p=0.03). Nutrition knowledge was higher in urban areas compared to rural areas. The chi-squared test showed a significant difference in nutrition knowledge in urban and rural areas (p=0.04). The result of the multivariate logistic regression model indicated that the type of residence, rural vs urban areas, had an inverse significant effect on food security after controlling for our variables. Syrian refugees in rural areas had 79.9 greater odds of being food insecure compared with urban areas (odds ratio: 0.201, 95% CI: 0.053-0.758, p=0.01).

Conclusions

Nutrition knowledge, English adequacy, and women's education may be less important than types of residence. The type of residence is a contributing factor to food insecurity in Syrian refugees residing in Florida. A larger sample size would allow a clearer understanding of the relation of our variables with food insecurity.

## Introduction

Syrian refugees are vulnerable families who have been forced to flee their homeland because of an ongoing war and war-related violence [1]. As of April 2019, the records of the United Nations High Commissioner for Refugees (UNHCR) showed that the total number of registered Syrian refugees was 5,648,002 [2]. The United States, as the largest resettlement country worldwide [3], had hosted 21,353 Syrian refugees since June 2011. Of these, 1154 individuals were initially assigned to the State of Florida, as per the report of the Refugee Processing Center (RPC) overviewed in April 2019 [4].

The United States has multiple governmental programs with the objective of encouraging newly resettled refugees to experience self-reliance in a short period of time. The government expects that self-sufficiency is achieved within eight months from arrival to the United States. The Cash and Medical Assistance programs, which are administrated by the Department of State Office of Refugee Resettlement, reimburse states with 100% of services provided to refugees during the first eight months of arrival [5].

Even though newly arrived refugees are supported with basic life needs during their initial stay in the United States, studies have shown that such a population is at risk of food insecurity [6,7]. Uncertain availability of nutritionally adequate and safe food for active life is defined as food insecurity. Food insecurity may be experienced due to socioeconomic parameters that may become barriers to accessing the quality of life-promoting resources among refugees in this developed country [6] or because food assistance becomes inadequate after the first eight months.

Immigrants to the United States might have had originally healthier dietary patterns compared to their adopted pattern after resettlement. In the United States, they might tend to increase their consumption of calorie-dense foods; they might become acculturated to poor eating habits due to their poor nutrition knowledge. The improvement of diet quality has been associated with the availability of nutrition knowledge, budgeting skills, and food resources in households with limited resources in the United States [8]. When the effects of food insecurity on health outcomes were examined, an association between nutrition knowledge and diet quality was reported. It was suggested that food insecurity is the moderator of this association [9].

Language and education may contribute to difficulties in navigating the US system and the US food-related environment [6]. Limited nutrition knowledge may be a non-economic challenge that becomes a barrier to accessing culturally appropriate food and health services [10]. Refugees who reported difficulties in navigating the food environment were more likely to have high food insecurity in the United States [6,11]. 

The RPC report indicated that only 0.03% of Syrian refugees spoke English upon arrival to the United States [4]. Less than 1% of Syrian refugees achieved a graduate level of education, 4.62% earned some university credits or university degrees, 1.92% finished technical school, and 10.15% completed high school [4]. Gender role is a cultural norm in Syria; the majority of Syrians believe that women are in need of men's protection [12]. Syrian men tended to be more educated than Syrian women; the literacy rate was 78% and 51% among Syrian males and Syrian females, respectively [13]. The majority of the arriving Syrian refugees in the United States were women and children [14]. Such findings combined with the statistical breakdown by RPC led to an expectation of a low education profile among Syrian women and poor English literacy in Syrian refugees upon arrival to the United States.

We hypothesized that food insecurity was going to be detected among Syrian refugees in Florida. English proficiency and women's education were proposed as predictors of food insecurity in this population. Although the literature did not provide information on nutrition knowledge in Syrian refugees, nutrition knowledge was proposed as a predictor of food insecurity as well [6,10,11]. The primary objectives were to measure food security in 80 Syrian refugee households residing in Florida and to determine whether English proficiency, women's education, and nutrition knowledge would be socioeconomic predictors of food insecurity among Syrian refugees. An abstract summarizing this study revealed that the type of residence is a contributing factor to food security in Syrian refugees residing in Florida [15]. A larger sample size would allow a clearer understanding of the relation of nutrition knowledge, English proficiency, and women's education with food security [15]. 

It is noteworthy to mention that this research is derived from a large dissertation project that investigated food security and the impact of various socioeconomic factors on food security among Syrian refugees residing in Florida [16]. Migration from Syria to the United States is a challenging experience due to the substantial differences in the structures and cultures of these two countries. The United States is a highly developed country compared with Syria which is a developing country. In addition, other differences such as cultural norms, demographic characteristics, and language spoken are considerable. Unfamiliarity with the US system, combined with socioeconomic differences, may create a cluster of challenges for Syrian refugees. Such challenges may contribute to food insecurity resulting in low quality of life and low economic contribution. On the other hand, determining food security within the context of these challenging additional factors may provide a better interpretation of their reality at the individual and community levels. Once factors are identified, appropriate interventions may be implemented to lessen food insecurity pre- or post-arrival in the United States [15,17,18].

## Materials and methods

The research model

**Figure 1:**
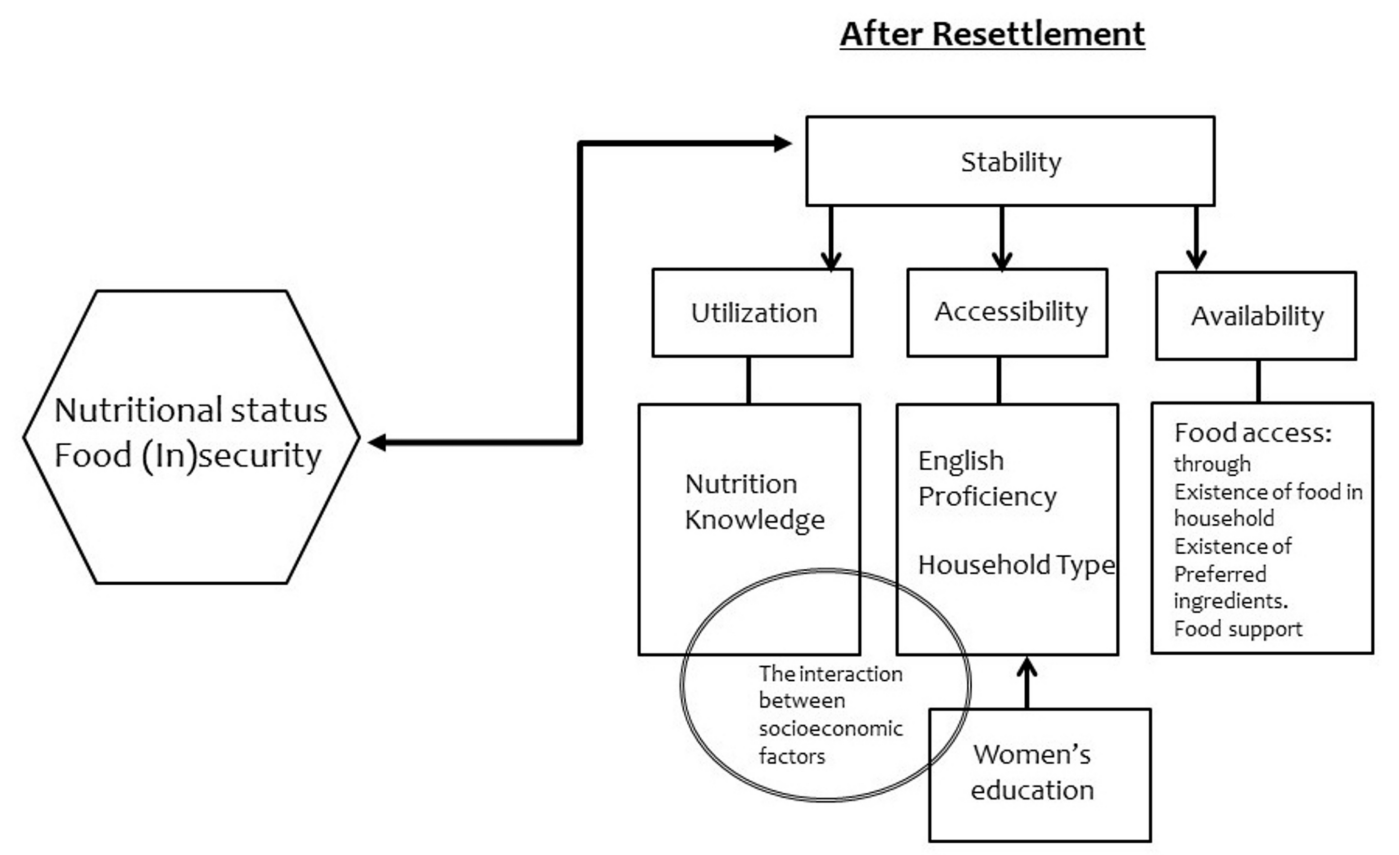
The model of food security and nutrition knowledge, English proficiency, and women's education among Syrian refugees in Florida

The food security and socioeconomic factors model for Syrian refugees in Florida was developed (Figure 1) as a result of merging three food insecurity models developed by three different organizations. The models used are the Interface between Food Insecurity and Violent Conflict by the Food and Agriculture Organization (FAO) of the United Nations [19], the Conceptual Framework of Food Security and Nutrition developed by InWent Capacity Building International, Germany, on behalf of the Federal Ministry of Economic Cooperation and Development [20], and the Conceptual Framework of the Nutritional Status at Household Level developed by Gross and colleagues in 2000 [21].

This particular research had a main concept that included food (in)security and nutrition knowledge among Syrian refugees. In reality, socio-demographic characteristics, including level of education and language literacy, contributed to difficulties in utilizing services among refugees living in the United States [22]. The effect of English proficiency and women's education on food insecurity was also examined in our research.

The core of our model consisted of four main constructs: Utilization, Accessibility, Availability, and Stability [20]. Utilization refers to the ability to purchase, prepare, and consume a balanced meal and depends on knowledge and habits. Thus, we included the variable of Nutrition Knowledge under the Utilization construct. The variable of English proficiency was utilized and categorized under the Accessibility construct, since this construct included resources of the social environment. Inadequate education led to inadequate care for women and children as per the United Nations Children's Fund (UNICEF) model for malnutrition released in 1992 [23]. Therefore, we utilized women's education as a contributing factor that might have an effect on Accessibility and Utilization in our model. Stability is a temporal dimension in food security and refers to the timeframe over which food security is sustainable. Stability occurs when consistency in availability, accessibility, and utilization is experienced.

In addition, other variables were considered during the course of data collection in order to measure food security and levels of food insecurity. Following the Food Security Core Model (FSM) by the United States Department of Agriculture (USDA), we included the following: prices of food, food access and availability of food in households, and equal distribution of meals among members of households [24]. These elements were included under the Availability construct. We also included Household Type in this research, which was listed under the Accessibility construct as a combination of the social and physical environment. Our objective with this inclusion was to determine the differences of food insecurity in different areas and cities of residence. 

Design

The study lasted for five months and was conducted in the State of Florida. It started on August 1, 2019, and ended on November 19, 2019. Two semi-structured interview questionnaires that aimed to measure food security and to assess nutrition knowledge were compiled and administered to Syrian refugees living in Miami, West Palm Beach, Orlando, and Tampa. The interviewer spoke Arabic, which was the native language of the interviewees. The approval of the Florida International University Institutional Review Board (FIU-IRB) was obtained as follows: the IRB protocol approval number is IRB-18-0301-CR01 and the TOPAZ reference number is 107023. English and Arabic versions of informed consent were developed and approved by FIU-IRB. 

The purpose of the research was communicated to the leaders of the Syrian immigrant community in Miami, Florida. The leaders of the Syrian immigrant community were initially responsible to assist Syrian refugee families with the resettlement process upon arrival in Miami. Initially, the leaders assisted the research team with the recruitment of Syrian refugees residing in Miami. They informed us that multiple Syrian refugee families had moved throughout Florida cities and to other states upon arrival. Thus, word of mouth was adopted eventually as another strategy to recruit our participants in West Palm Beach, Orlando, and Tampa. Our recruitment process revealed that a total of 85 Syrian refugee households were residing in Miami, West Palm Beach, Orlando, and Tampa. Tampa residents were mainly located in rural areas, while those in Miami, West Palm Beach, and Orlando were mainly urban dwellers. 

When the purpose of the research was communicated to such households, five households disagreed to participate. Our research included 80 households who met the inclusion criteria and agreed to be interviewed. The inclusion criteria were households of displaced Syrians in Florida who were originally registered by the United Nations as refugees and resettled in the United States after the beginning of the Syrian war in 2011. Our exclusion criteria were Syrian immigrants who arrived in Florida before 2011, displaced Syrians who arrived in Florida after 2011 but were not registered by the United Nations, Syrian immigrants with different visa documentations, and asylum seekers with Syrian nationality residing in Florida. Possibly, Syrian refugee families might have moved throughout Florida cities and to other states upon arrival. 

Semi-structured interview questionnaires

Food security, nutrition knowledge, English proficiency, and women's education in participating households were collected. Multiple questionnaires were compiled into a comprehensive questionnaire with the objective of measuring food insecurity and nutrition knowledge (Appendix A). As part of the demographic characteristics, information about English proficiency and women's education was collected. Gender, city of residence in Syria, location of the transitional period, month and year of arrival to the United States, type of households, number of children, and employment status were collected. In one-on-one sessions, the comprehensive questionnaire was completed in an average of 45 minutes per session. Since the sessions took place in person, the questionnaires triggered further explanations from interviewees. Comments and information obtained were documented for future qualitative analysis. We also obtained data on other variables that were involved in our model to be examined and analyzed based on conclusions drawn from a comprehensive literature review.

Food security

The FSM-USDA model was adopted; it included 15 questions with an additional three subsequent questions providing a comprehensive understanding about food intake and food supply of households during the past 12 months (Appendix B) [24]. The questions covered the behavioral and psychological responses to circumstances of food shortage or insufficient financial resources.

The construct of questions directed interviewees to answer either affirmatively or negatively. The three options of answers were always true, often true, or never true besides the option of a refusal to answer. In addition, subsequent questions aimed to detect frequencies of events, if responses were affirmative. For instance, an affirmative response to an adult having to cut or skip meals due to insufficient food was followed by a question about the frequency of occurrence, two months or less or three months and more during the past 12 months.

Following the scaling system of the FSM-USDA model [24], every affirmative response of either always true or sometimes true was given 1 point. A total score of 10 points was given to households without children, and a total score of 16 was given to households with children. Food insecurity was classified into three levels of severity; a greater number of affirmative responses indicated greater severity of food insecurity. In all of the households, a score of ≤2 was classified as food secure. In households with children, a score of ≥3 to ≤7 was classified as food insecure without hunger, a score of ≥8 to ≤12 was classified as moderate food insecure with hunger, and a score of >12 was classified as severe food insecure with hunger. In households without children, a score of ≥3 to ≤5 was classified as food insecure without hunger, a score of ≥6 to ≤8 was classified as moderate food insecure with hunger, and a score of >8 was classified as severe food insecure with hunger.

Nutrition knowledge

The questionnaire was adopted from a study that aimed to measure nutrition knowledge, dietary behavior, and nutrient intakes of newly arrived Hispanic adolescent females in the United States by Parga [25]. The original questionnaire included 30 items to assess different aspects of nutrition knowledge including healthy eating habits, vitamins and minerals, dietary intake in relation to chronic diseases, and the individual's perception toward body image and weight change status.

This questionnaire was modified to simplify questions about the nutrition concepts that were assessed. Vitamins were identified with their simple alphabetical names instead of generic names, vitamin C replaced ascorbic acid, vitamin B1 replaced thiamin, and vitamin B2 replaced riboflavin.

Questions regarding body image and weight changes tended to be subjective in nature; they were excluded from the nutrition knowledge assessment. Responses to such questions were obtained, because they might allow the use of weight change as a confounding variable in the context of acculturation and health status in future research.

The questionnaire was translated and back-translated, English to Arabic and Arabic to English, by two bilingual Syrian immigrants to ensure the accuracy of translation. In order to validate the cultural appropriateness, a pilot study was conducted through voluntary participation of six Syrian immigrants who arrived in the United States after 2011. The result of the pilot study showed that newly arrived Syrians might be unfamiliar with Thousand Island dressing although they might consume alternative products. The general term of creamy salad dressing was used among the choices given to assess knowledge of high-fat food. 

Seven questions on healthy diet content, sources of macronutrients, adolescent nutrition, and alcohol intake were utilized to assess healthy eating practices [25]. Questions to assess knowledge on vitamins and minerals included 10 questions about the toxicity of vitamin supplements, the function of antioxidants, and the best sources of the following nutrients: calcium, iron, vitamin D, vitamin B12, and folic acid. Knowledge of dietary intake in relation to chronic diseases was assessed with a total of eight questions: two questions on fiber intake and the function of fiber, two questions on fat intake and obesity, and four questions on different types of fat and prevention of heart disease. A score of 4 points was assigned to a question with a correct answer, and a score of 0 was given to incorrect answers or refusal to answer. A total of 100 points was the maximum possible score. A score in the average between 0 and ≤25 was considered poor nutrition knowledge, a score of 26 and ≤50 was considered fair nutrition knowledge, a score of 51 and ≤75 was considered good nutrition knowledge, and a score of >75 was considered high nutrition knowledge.

English proficiency

In the demographic section of the compiled questionnaire, a self-rating for the four components of English proficiency was requested. Components of writing, reading, speaking, and comprehension were rated as poor, fair, good, and fluent. Afterward, a rate of fair or greater in two components was classified as adequate English. English proficiency was measured as adequate English and inadequate English according to the result of the self-rating by the participants.

Women's education

In the demographic section of the compiled questionnaire, the academic level of women in households was questioned. Level of education was classified into three categories: (1) incomplete high school, (2) completed high school, or (3) greater than high school. The first category, incomplete high school, included women who reached high school level but did not earn the diploma, those who finished intermediate school, and women who finished primary school. The second category included women who completed high school and earned a high school diploma, and the third category included those who reached university level and/or women who earned a university degree.

Statistical analysis

SAS Studio University Edition was used for all statistical analyses. Descriptive statistics, one-way frequency, and table analysis were used to identify Syrian refugees with regard to demographic characteristics and variables of interest. Fisher's exact test and chi-squared tests were utilized to determine the association between food security, levels of food insecurity, and predictors of interest in urban and rural areas as well as in cities of residence. Similarly, the associations between food security status and our predictors were determined by applying Fisher's exact test and chi-squared test. The predictors were nutrition knowledge, English proficiency, and women's education. Binary logistic regression and an interaction plot were conducted to examine the effects of predictors on food security status in rural and urban areas and in cities of residence.

## Results

Table 1 demonstrates the selected demographic characteristics of our participants including gender, nutrition knowledge, English proficiency, and women's education.

**Table 1:** Demonstration of selected demographic characteristics of the participants including gender, nutrition knowledge, English proficiency, and women's education (%) Column-based percentages within a specific category

Characteristic	n (%)
Gender of respondents	
Female	63 (78.7)
Male	17 (21.3)
Type of households	
Households with children	71 (88.7)
Households without children	9 (11.3)
Nutrition knowledge	
Respondent scored poor	10 (12.5)
Respondent scored fair	46 (57.5)
Respondent scored good	24 (30.0)
English proficiency	
Respondent rated ≥ fair in reading	16 (20.0)
Respondent rated ≥ fair in writing	16 (20.0)
Respondent rated ≥ fair in speaking	13 (16.3)
Respondent rated ≥ fair in comprehension	19 (23.8)
English proficiency/adequacy	
Respondent with adequate English	20 (25.0)
Respondent with inadequate English	60 (75.0)
Women's education	
Women with incomplete high school	61 (76.3)
Women with complete high school level	11 (13.7)
Women with some university education/university degree	8 (10.0)

Food security

Of the 80 households, 20% were food secure, while 80% of households experienced food insecurity at different levels, and the mean of the FSM-USDA score was 4.7±2.6. Households of Syrian refugees in rural areas (n=43) were moderately food insecure with hunger (5.00±2.4), and Syrian refugees in urban areas (n=37) were food insecure without hunger (4.50±2.8). Fisher's exact test showed significant differences between the levels of food insecurity in rural and urban areas (p=0.02) (Table 2).

**Table 2:** Levels of food insecurity by types of residence **Fisher's exact test. Statistically significant, p=<0.05 (%) Column-based percentages within a specific category

Levels of food insecurity	Rural areas n (%)	Urban areas n (%)	P-value
Number of respondents (n)	43	37	
Food security	4 (9.3)	12 (32.4)	0.02**
Food insecurity without hunger	31 (72.1)	18 (48.6)	
Moderate food insecurity with hunger	8 (18.6)	5 (13.5)	
Severe food insecurity with hunger	0	2 (5.5)	

When households were categorized into food secure and food insecure in the two different settings, there were also significant differences between food security status among households when tested by types of residence and city of residence (p=0.009 and p=0.02, respectively) (Table 3).

**Table 3:** Food security status in relation to types of residence and city of residence *Chi-squared test. **Fisher's exact test. Statistically significant, p=<0.05 (%) Column-based percentages within a specific category

Variables	Number of respondents (n)	Food-secure households n (%)	Food-insecure households n (%)	P-value
Number of households	80	16 (100.0)	64 (100.0)	
Types of residence				0.009*
Rural areas	43	4 (25.0)	39 (60.9)	
Urban areas	37	12 (75.0)	25 (39.1)	
City of residence				0.02**
Miami	18	5 (31.25)	13 (20.4)	
West Palm Beach	10	5 (31.25)	5 (7.8)	
Orlando	9	2 (12.5)	7 (10.9)	
Tampa	43	4 (25.0)	39 (60.9)	

Similarly, Fisher's exact test showed there were significant differences in the levels of food insecurity in the four cities (p=0.04) (Table 4).

**Table 4:** Levels of food insecurity of households by city of residence **Fisher’s exact test. Statistically significant, p=<0.05 (%) Column-based percentages within a specific category

Levels of food insecurity	Miami n (%)	West Palm Beach n (%)		Orlando n (%)	Tampa n (%)	P-value
Number of respondents (n)	18		10	9	43	
Food security	5 (27.8)		5 (50.0)	2 (22.2)	4 (9.3)	0.04**
Food insecurity without hunger	9 (50.0)		3 (30.0)	6 (66.7)	31 (72.1)	
Moderate food insecurity with hunger	3 (16.7)		1 (10.0)	1 (11.1)	8 (18.6)	
Severe food insecurity with hunger	1 (5.5)		1 (10.0)	0	0	

**Figure 2:**
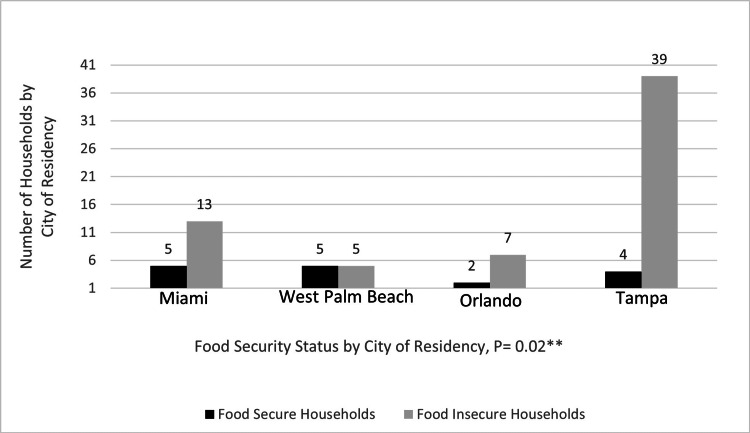
The distribution of food security and food insecurity among households by city of residence **Fisher's exact test. Statistically significant, p=<0.05

Figure 2 shows the distribution of food security among the cities, and it shows that Tampa had the highest number of food-insecure households. Fisher's exact test showed significant differences in food security status among households in the four cities (p=0.02).

Nutrition knowledge

Among the total households, 10 (12.5%) had poor nutrition knowledge, 46 (57.5%) had fair nutrition knowledge, and 24 (30%) scored good nutrition knowledge. The mean nutrition knowledge score (42.0±13.6) indicated that Syrian refugees had fair nutrition knowledge. The chi-squared test showed a significant difference in nutrition knowledge in urban and rural areas (p=0.04) (Table 5). It also showed a significant difference in nutrition knowledge in the participating households in the four cities (p=0.02) (Table 6). Fisher's exact test showed that there were no significant differences in food security status among households with different nutrition knowledge (p=0.6) (Table 7).

**Table 5:** Description of the participants by types of residence *Chi-squared test. **Fisher's exact test. Statistically significant, p=<0.05 (%) Column-based percentages within a specific category

Characteristics	Rural areas n (%)	Urban areas n (%)	P-value
Number of respondents (n)	43	37	
Nutrition knowledge			0.04*
Respondent scored poor	3 (7.0)	7 (19.0)	
Respondent scored fair	30 (70.0)	16 (43.2)	
Respondent scored good	10 (23.3)	14 (37.8)	
English proficiency/adequacy			0.2**
Respondent with adequate English	8 (18.6)	12 (32.4)	
Respondent with inadequate English	35 (81.4)	25 (67.6)	
Women's education			0.03*
Incomplete high school	37 (86.0)	24 (64.9)	
Complete high school and greater	6 (14.0)	13 (35.1)	

**Table 6:** Description of the participants by city of residence *Chi-squared test. **Fisher's exact test. ***One-way ANOVA test. Statistically significant, p=<0.05 (%) Column-based percentages within a specific category

Characteristics	Miami n (%)	West Palm Beach n (%)	Orlando n (%)	Tampa n (%)	P-value	
Number of respondents (n)	18	10	9	43		
Nutrition knowledge					0.02*	
Respondent scored poor	4 (22.2)	3 (30.0)	0	3 (7.0)		
Respondent scored fair	5 (27.8)	4 (40.0)	7 (77.7)	30 (70.0)		
Respondent scored good	9 (50.0)	3 (30.0)	2 (22.3)	10 (23.3)		
English proficiency					0.1**	
Respondent with adequate English	8 (44.5)	3 (30.0)	1 (11.1)	8 (18.6)		
Respondent with inadequate English	10 (55.5)	7 (70.0)	8 (88.9)	35 (81.4)		
Women's education					0.07***	
Incomplete high school	10 (55.6)	8 (80.0)	6 (66.7)	37 (86.0)		
Complete high school and greater	8 (44.4)	2 (20.0)	3 (33.3)	6 (14.0)		

**Table 7:** Food security status in relation to variables of interest *Chi-squared test. **Fisher's exact test. Statistically significant, p=<0.05 (%) Column-based percentages within a specific category

Variables	Food-secure households n (%)	Food-insecure households n (%)	P-value
Number of households	16 (100.0)	64 (100.0)	
Nutrition knowledge			0.6**
Poor	3 (18.75)	7 (10.9)	
Fair	8 (50.0)	38 (59.4)	
Good	5 (31.25)	19 (29.7)	
English proficiency/adequacy			1.0**
Inadequate	12 (75.0)	48 (75.0)	
Adequate	4 (25.0)	16 (25.0)	
Two levels of women's education			1.0**
Incomplete high school	12 (75.0)	49 (76.6)	
Complete high school	4 (25.0)	15 (23.4)	
Three levels of women's education			0.8**
Incomplete high school	12 (75.0)	49 (76.6)	
Complete high school	3 (18.7)	8 (12.5)	
Some university education/university degree	1 (6.3)	7 (10.9)	

English proficiency

One-way frequency showed that 60 (75%) of households had inadequate English and 20 (25%) had adequate English. In rural areas, the frequency of inadequate English was 35, which accounted for 81.4% of households in these areas. English proficiency was significantly different in households in rural areas (Student t-test: p=0.003). Only eight households (18.6%) had adequate English in rural areas. Again, the frequency of households with inadequate English was higher than the frequency of adequate English in urban areas, 25 (67.57%) versus 12 (32.43%) (Student t-test: p=0.0002). Fisher's exact test showed that there were no significant differences in English proficiency in rural and urban areas (p=0.2) (Table 5). When one-way frequency for English proficiency was categorized based on city of residence, the frequency of adequate English was 8 (44.4%) in Miami, 3 (30%) in West Palm Beach, 1 (11.1%) in Orlando, and 8 (18.6%) in Tampa. Fisher's exact test did not show that there were significant differences in English proficiency in different cities (Table 6). Households were grouped into households with adequate English (n=20) and households with inadequate English (n=60); Fisher's exact test did not show significant differences in food security status among households with different English adequacy (Table 7).

Women's education

Around 23.7% of Syrian refugee women had an education level of high school diploma or higher, while 76.3% did not complete their high school education. When categorized by types of residence, the percentage of women who completed high school was higher in urban areas compared with rural areas (35.14% versus 13.95%). The chi-squared test showed that there were significant differences in the levels of women's education in rural and urban areas (p=0.03) (Table 5). Based on the city of residence, the percentages of households with women who completed high school were 44.4%, 20%, 33.3%, and 13.95% in Miami, West Palm Beach, Orlando, and Tampa, respectively. The differences in women's education in different cities were marginally significant based on one-way non-parametric ANOVA (p=0.07) (Table 6). Nevertheless, Fisher's exact test did not show significant differences either in food security status or in the levels of food insecurity when households were categorized by two levels of women's education (p=1.0) (Table 7). Afterward, households were categorized into three classes of women's education: women with incomplete high school, women who completed high school education, and those with some university education/university degree. Households with women's education of some university education/university degree, completed high school, and incomplete high school constituted 10% (n=8), 13.75% (n=11), and 76.25% (n=61), respectively, of our sample. Fisher's exact test did not result in significant differences in food security status among households categorized by three classes of women's education (Table 7).

Women's education and English proficiency

It is noteworthy to mention that the percentage of women with adequate English was higher among women in the group of completed high school than in the group of women with incomplete high school (63.16% versus 13.11%). Among women with complete high school education, there were 12 (63.16%) with adequate English and seven (36.84%) with inadequate English. In contrast, the frequency of inadequate English was greater than the frequency of adequate English in women with incomplete high school (n=53 (86.89%) versus n=8 (13.11%)). Fisher's exact test showed that there were significant differences between the two groups (p≤0.0001). This led to examining the association of food security with women's education and with English proficiency. When English adequacy was controlled, one-way frequency showed that the total number of households with adequate English was 20. There were no significant differences in food security status in the two groups of households (Table 8). 

**Table 8:** Food security status in households with different levels of women's educations along with controlling English proficiency *Chi-squared test. **Fisher's exact test. Statistically significant, p=<0.05 (%) Column-based percentages within a specific category

Variables	Food-secure households n (%)	Food-insecure households n (%)	P-value
Number of households with adequate English	4 (100.0)	16 (100.0)	
Three levels of women’s education			0.8**
Incomplete high school	1 (25.0)	7 (43.75)	
Complete high school	2 (50.0)	5 (31.25)	
Some university education/university degree	1 (25.0)	4 (25.0)	
Two levels of women's education			0.6**
Incomplete high school	1 (25.0)	7 (43.75)	
Complete high school and higher	3 (75.0)	9 (56.25)	

Regression models

The results of multivariate logistic regression models showed that types of residence had an inverse significant effect on food security, which remained significant after controlling for English proficiency, nutrition knowledge, and women's education (Table 9). These results revealed that Syrian refugees in rural areas had a 79.9% more chance of being food insecure compared with urban areas (odds ratio: 0.201, 95% CI: 0.053-0.758, p=0.01) (Table 9).

**Table 9:** Multivariate logistic regression demonstrating the effect of selected variables on food security status in participating households by types of residence Statistically significant, p<0.05 (-): reference group; β: estimate; B: odds ratio; SE: standard error

	Model 1				Model 2			
Covariate	β	B	SE	P-value	β	B	SE	P-value
Constant	-0.7		0.35	0.03	-0.8		1.01	0.4
Types of residence								
Rural areas	-1.5	0.214	0.63	0.01	-1.6	0.201	0.67	0.01
Urban areas	0	-	-		0	-	-	
Nutrition knowledge								
Good					-0.26	0.77	0.93	0.8
Fair					-0.18	0.83	0.86	0.8
Poor					0	-	-	-
English proficiency								
Inadequate					0.11	1.118	0.83	0.9
Adequate					0	-	-	
Women's education								
Incomplete high school					0.26	1.302	0.79	0.7
Complete high school					0	-	-	

Nevertheless, all four cities showed significant effects on food security status in households of Syrian refugees; Tampa refugees had significantly higher food insecurity compared with West Palm Beach and other cities (odds ratio: 0.103, 95% CI: 0.020-0.514, p=0.006). Therefore, Tampa had an 89.7% greater chance of having food-insecure households compared to other cities (Table 10, Model 1). After adjusting for covariates, Tampa was the only city with a high probability of food insecurity among refugees when compared with West Palm Beach, Tampa refugees were 7.4 times more likely to be food insecure, and refugees living in Tampa had a 90.4% higher risk of being food insecure than in other Florida cities (odds ratio: 0.096, 95% CI: 0.017-0.530, p=0.007) (Table 10, Model 2).

**Table 10:** Multivariate logistic regression demonstrating the effect of selected variables on food security status in participating households by city of residence Statistically significant, p=<0.05 (-): reference group; β: estimate; B: odds ratio; SE: standard error

	Model 1				Model 2			
Covariate	β	B	SE	P-value	β	B	SE	P-value
Constant	1.2		0.63	1	-0.29		1.18	0.8
City of residence								
Miami	-0.9	0.385	0.8	0.3	-0.9	0.413	0.85	0.3
Orlando	-1.3	0.286	1.02	0.2	-1.3	0.267	1.09	0.2
Tampa	-2.3	0.103	0.82	0.006	-2.3	0.096	0.87	0.007
West Palm Beach	0	-	-		0	-	-	
Nutrition knowledge								
Good					-0.04	0.965	0.97	1.0
Fair					0.05	1.059	0.94	1.0
Poor					0	-	-	
English proficiency								
Inadequate					0.27	1.307	0.85	0.8
Adequate					0	-	-	
Women's education								
Incomplete high school					0.09	1.105	0.01	0.9
Complete high school					0	-	-	

**Figure 3:**
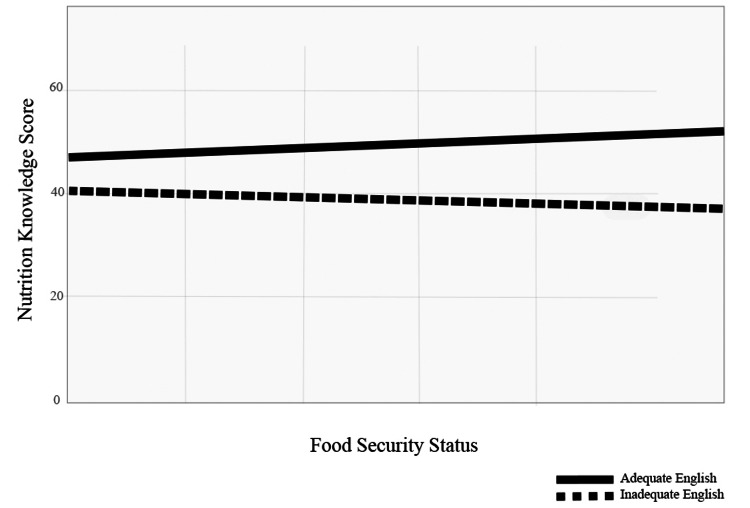
The interaction between food security, nutrition knowledge, and English proficiency among all of the households

Lastly, an interaction plot that included a nutrition knowledge score, English adequacy, and food security suggested that food security would be more likely to occur when households had a higher score in nutrition knowledge and greater English proficiency (Figure 3).

**Figure 4:**
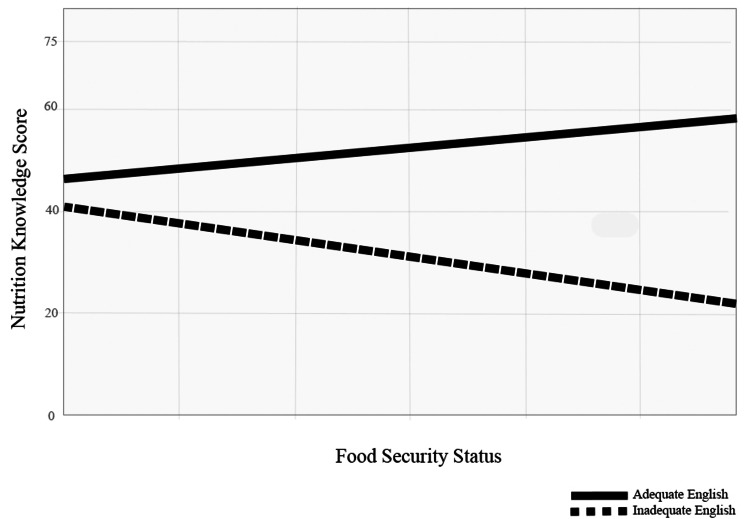
The interaction between food security, nutrition knowledge, and English proficiency in households residing in urban areas

Moreover, when we grouped fair and good nutrition knowledge and compared them to poor, the interaction plots with these variables confirmed that households with greater nutrition knowledge and greater English proficiency are more likely to experience food security in urban areas (Figure 4).

## Discussion

Food security

Since the beginning of the war in Syria, the overall number of Syrian refugees who were initially assigned to the State of Florida was 1154, as per the Department of State RPC [4]. The Inter Press Service organization represented the Syrian refugee families in Southern Florida as an underserved refugee community that included 90 individuals [26]. Possibly, Syrian refugee families might have moved throughout Florida cities and to other states upon arrival. Our research included 80 households that comprised 360 Syrian refugees residing in Florida.

Food insecurity is usually observed among refugees based in the United States over a long period of time, which may cause poor health outcomes and health disparities [7]. In 2011, it was found that 85% of the refugees living in the Northeastern United States region experienced food insecurity compared to the national average of 14% [11]. Our findings showed that food insecurity was frequent (80%) among Syrian refugees residing in Florida. Environmental factors, including types of residence such as rural or urban, may affect food security status at the household level. These findings are congruent with those in the general population as food insecurity is more prevalent in the rural areas compared with the urban areas in the United States [27].

The US Economic Research report completed in 2016 showed that the prevalence of food insecurity was 15.4% in rural areas, while it was 14.1% in urban areas. The report suggested that geographic location is an important environmental factor that affects food security. Based on these reports from the literature, we included this factor in our evaluation when developing an intervention to promote quality access to nutritious food [28]. In our research, 90.7% of households in rural areas were food insecure compared with 67.6% of food-insecure households in urban areas. Statistically, there were significant differences in food security among households in different areas and cities.

Nutrition knowledge 

The average score of nutrition knowledge indicated that most participants had fair nutrition knowledge. Although there were no significant differences in food security status in households with different scores of nutrition knowledge, nutrition knowledge was significantly different in households in different cities and types of residence (Table 5 and Table 6). Our interaction plot also demonstrated that there was a positive relationship between the nutrition knowledge score and food security status in all households (Figure 3).

Despite that, to the best of our knowledge, no research has been conducted to examine the effect of nutrition knowledge in combating food insecurity among Syrian refugees in the United States. The literature confirms that nutrition knowledge and nutrition education are barriers to food security among refugees. The Office of Refugee Resettlement of the US Department of Health and Human Services also incorporated nutrition education into the components of the Newly Arrived Refugees program to increase the knowledge about USDA dietary guidelines [10]. In 2013, the National Research Council, Institute of Medicine, and Food and Nutrition Board suggested that nutrition-related support might reduce food insecurity if tailored to the geographical location and circumstances of individuals [29]. Therefore, nutrition knowledge has an effect on food security, and poor nutrition knowledge is a risk factor for food insecurity in our population. Greater nutrition knowledge increases the likelihood of food security. A larger sample size would have supported our finding by improving the statistical power.

Other studies have utilized different tools to measure nutrition knowledge when investigating food security; therefore, our findings may be difficult to compare with others that have consistent results. A large study funded by the National Cancer Institute that involved 1874 households proposed nutrition education as an intervention against food insecurity in low-income families [30]. Researchers concluded that lack of nutrition knowledge is one of the reasons for the higher frequency of unhealthy behaviors in food-insecure households. In this particular study, food security was examined in relation to dietary habits using the Food Habits Questionnaire (FHQ). Despite some discrepancies with our tool and FHQ, the two measurement tools aimed to assess the same nutrition-related concepts. For instance, our tool included a question about the recommended servings of fruits and vegetables per day, while the FHQ tool was to assess servings of fruits and vegetables usually consumed per day in the past year. This assessment tool likely reflected the nutrition knowledge of participants, food security was measured using FSM-USDA, and participants were of low income and at risk for food insecurity in the United States. Therefore, their findings strongly support our conclusions that increasing nutrition knowledge is a food security-promoting strategy in the Syrian refugee community.

English proficiency

The majority of our respondents did not have adequate English proficiency (75%). This percentage increased in rural areas to 81.4%, whereas it was less in urban areas (67.6%). Statistically, we failed to prove our hypothesis that households with fair or fluent English proficiency were less likely to be food insecure. This was in contrast to a study that associated English difficulty with higher food insecurity among 281 refugees who resettled in the United States, independent of the level of income [6].

In fact, only 0.03% of Syrian refugees admitted to the United States spoke English [4]. An access to English learning classes is a service offered by refugee resettlement agencies and is reimbursed by the Department of State as a division of the Cash and Medical Assistance program [31]. Our small sample size might have statistically reflected reality. When English proficiency was included in the interaction plot with the nutrition knowledge score and food security status, the result decently showed that greater English adequacy with a greater nutrition knowledge score promoted higher food security status (Figure 3 and Figure 4). A study supported our interaction plot-based conclusion and concluded that nutrition knowledge deficit and English proficiency were barriers in a group of 40 refugees that included Iraqi refugees who resettled to the United States [32]. In fact, Iraq and Syria are neighboring countries in the region of the Middle East; Iraqi and Syrian individuals may share a variety of similar norms in terms of demographics, food-related culture, and native language. Hence, our hypothesis of associating a higher score in nutrition knowledge and fair or fluent English proficiency with an increased likelihood of food-secure households was accepted.

Women's education

In our study, the varying levels of women's education among participating households hindered the ability to achieve statistically significant results. The overall number of households with women in the group that had completed high school was 19 (23.75%), and the overall number of households with women in the group that had not completed high school was 61 (76.25%). Table 7 led us to conclude that the ratio of the households with women with incomplete high school education to the households with women with complete high school education was 3:1. This ratio remained constant when these groups of households were categorized by food security status. In other words, there were three households with women with incomplete high school education for every household with a woman with complete high school education when categorized by food security.

This was in contrast to studies that showed a positive relationship between education and food security. A significant relationship was evident between food security and the level of education of 1847 respondents, of which women accounted for 85% of total respondents [30]. In this study, a greater education level was observed among food-secure households compared with food-insecure households. The percentages of different education levels in secure households were as follows: 34% with incomplete high school education, 31% with complete high school education, and 35% with some university education or a university degree. Thus, the distribution of different levels of education was nearly equated in the households that participated resulting in significant statistical power.

In our study, the percentages of education levels of women in food-secure households were as follows: 75% of women with incomplete high school education, 18.75% of women with complete high school education, and 6.25% of women with some university education or a university degree. Our statistical analysis challenged us to prove the definitive association. Our sample size limited the opportunity to have a better distribution of different education levels. Moreover, the report of RPC indicated that the vast majority of Syrian refugees admitted to the United States had incomplete high school education [4]. Approximately half of Syrian refugees (56%) completed primary and intermediate school. But only 10% completed secondary school or high school education. Hence, 66% of Syrian refugees had an education in high school or lower levels. When Syrian refugees were classified by gender, 47.9% were women [4]. Thus, the percentage of Syrian refugee women with incomplete high school education admitted to the United States during the course of our research would be approximately 72.5%. Obtaining a better distribution of education levels among Syrian refugee women in the United States may not be realistic. Therefore, the hypothesis that households with women with an education level of high school diploma or higher are more likely to be less food insecure was not fully accepted, and a larger sample size may not necessarily support our hypothesis or conclusion in the meantime.

Translation of findings into our research model

Food access, equal distribution of meals, and food price constituted the Availability construct in our model. Based on the result of multiple items of the FSM-USDA questionnaire, we affirmed that the construct of Availability was not consistent and its sustainability was less likely to happen. This inconsistency led to a negative impact on the Stability construct, which had a direct relationship with our main outcome, Food Security Status.

The Stability construct had a direct interchangeable interaction with the construct of Accessibility, which was English proficiency in this particular research. By applying our findings, we concluded that the effect of Accessibility on Stability was not observed statistically, but the interaction was evident in our interaction plot. Moreover, the direct interaction between women's education and Accessibility was observed. Women with complete high school education were more likely to have adequate English. Another variable we could have listed under Accessibility was the type of residence. Our findings showed that food security was significantly different in different cities and different residences and the type of residence might be a predictor of food security in this population. 

Lastly, the impact of the Utilization construct on Stability and the interaction between Utilization and Accessibility were confirmed. The association between English proficiency and nutrition knowledge was statistically significant. The interaction plot showed a clear explanation of the positive relation between the nutrition knowledge score and food security when English proficiency increased. This translation allows us to suggest that our model is likely applicable among Syrian refugees living in the United States if future research is of interest.

The strength of this research was the ability to create a clear insight about the experiences of Syrian refugees in Florida. The outcomes of our measures raised the awareness of the socioeconomic challenges that Syrian refugees may face in the United States. Such results may direct us to develop appropriate interventions among Syrian refugees in future research. The demographic characteristics of the researcher facilitated the recruitment process and accelerated the phase of data collection. The researcher was born in Syria and was fluent in Arabic. She was familiar with Syrian culture and the different norms of different Syrian cities. Thus, a trustful rapport was established with the participants, which reduced the bias associated with self-reporting and provided the opportunity to collect additional qualitative data.

The main limitation of this research was the sample size that prevented us from detecting significant associations statistically with regard to the education levels of women and English proficiency. Classification of our participants by city of residence further magnified the limitation created by our small sample size. Based on the power analysis, the number of families we were able to recruit was sufficient. However, we wished to tease out some of the more important variables in the study that we encountered such as families in rural versus urban areas. This created a challenge to establish power in some of our analyses. Future research should take these differences into account when establishing power regarding these variables. A larger sample size would allow a clearer understanding of the relation of our variables with food security and suggest remedial action.

## Conclusions

Most households (80%) of Syrian refugees who participated in this research were food insecure. Levels of food insecurity were greater in rural areas compared with urban areas. The difference was that in the rural areas, we observed more food insecurity with hunger compared with food insecurity without hunger in the urban areas. Most Syrian refugees had fair nutrition knowledge, but it was significantly different among cities as well as in rural and urban areas. Refugees in Tampa had lower food security; living in rural Tampa lowered the likelihood of having food security among Syrian refugees compared with other cities in Florida. English proficiency, nutrition knowledge, and women's education may be less important than location in being food secure in this population. Despite not finding statistical differences for those variables on food security, the results from the interaction plots suggest a route for future research with a larger sample size on the situation of Syrian refugees and what are the points of intervention to ameliorate their challenging situation. Moreover, future research should also address the status of this population, their lives, and their acculturation at periodic intervals in the United States. 

## Figures and Tables

**Figure 5:**
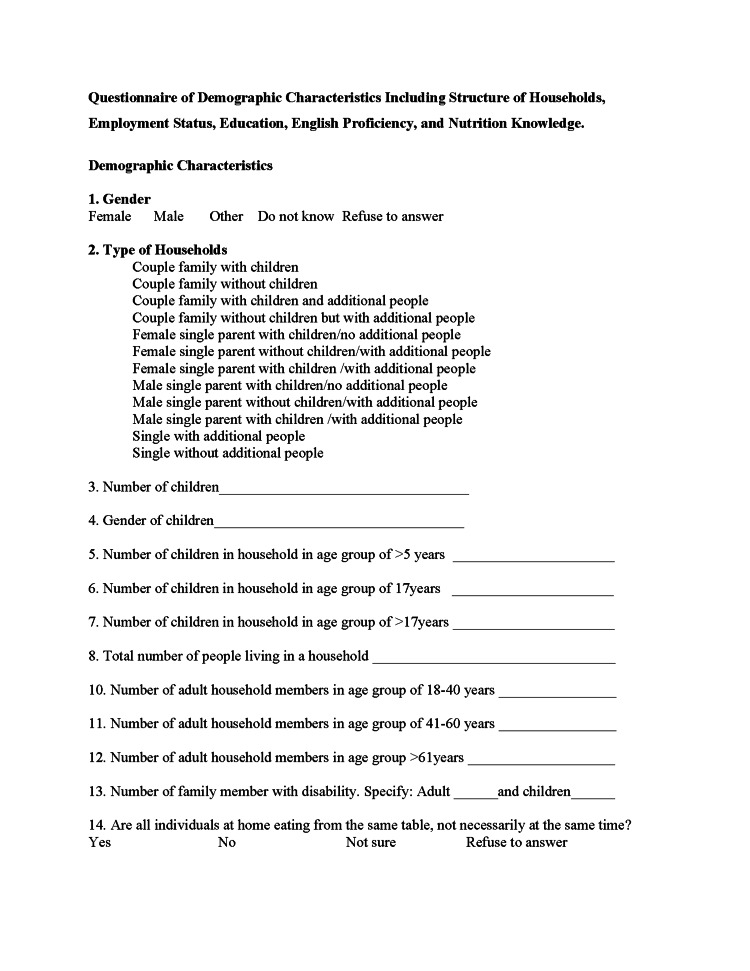
Comprehensive questionnaire

**Figure 6:**
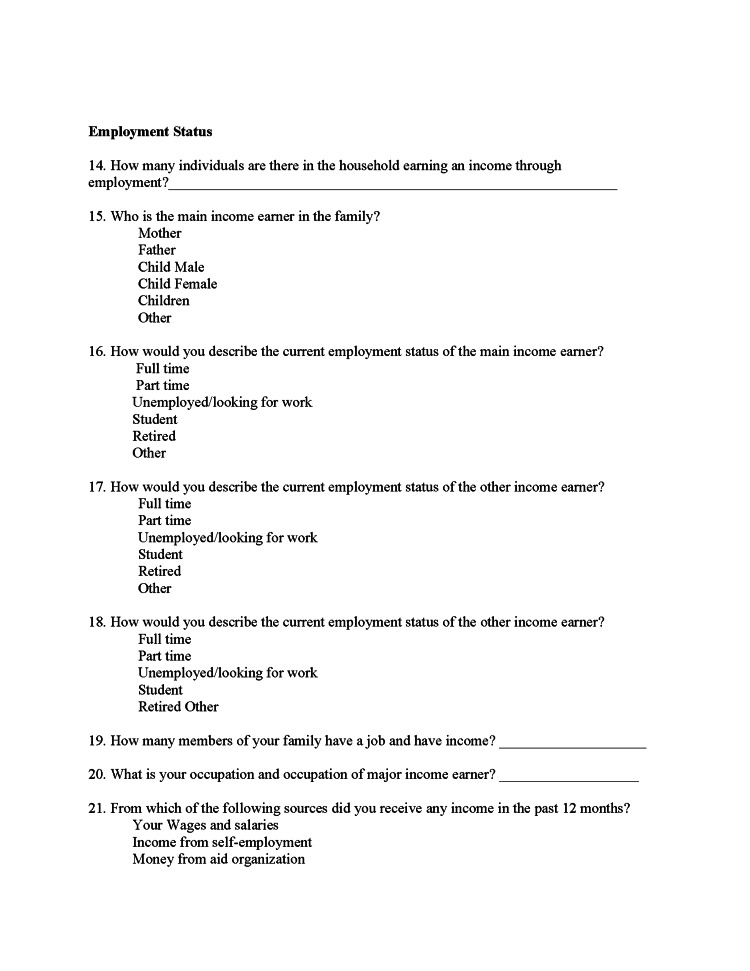
Comprehensive questionnaire (Contd.)

**Figure 7:**
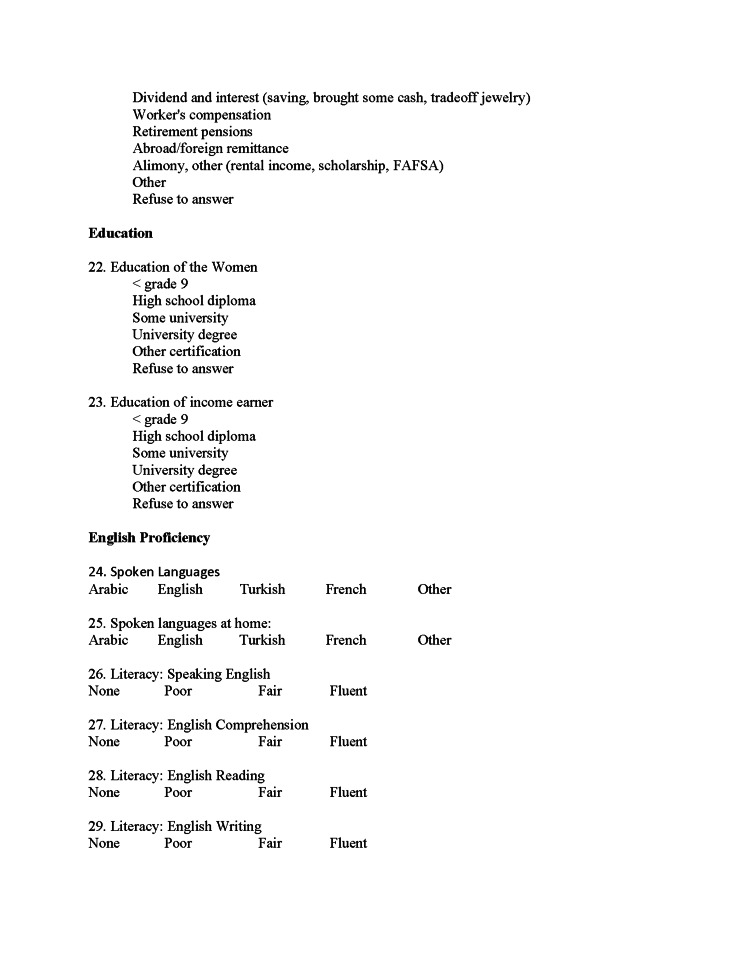
Comprehensive questionnaire (Contd.)

**Figure 8:**
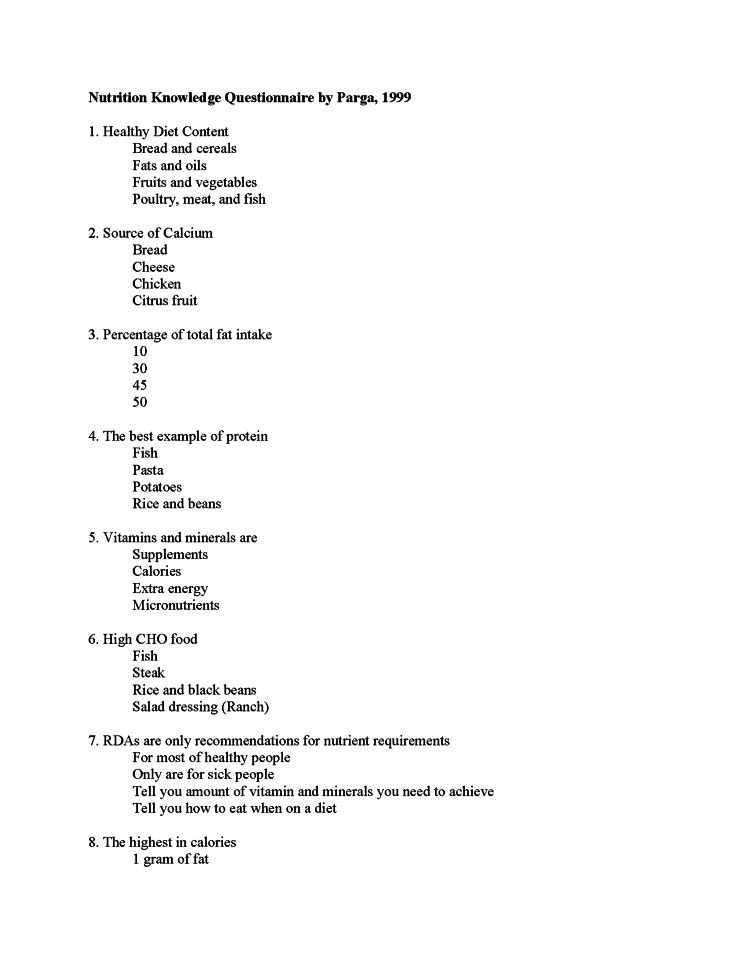
Comprehensive questionnaire (Contd.)

**Figure 9:**
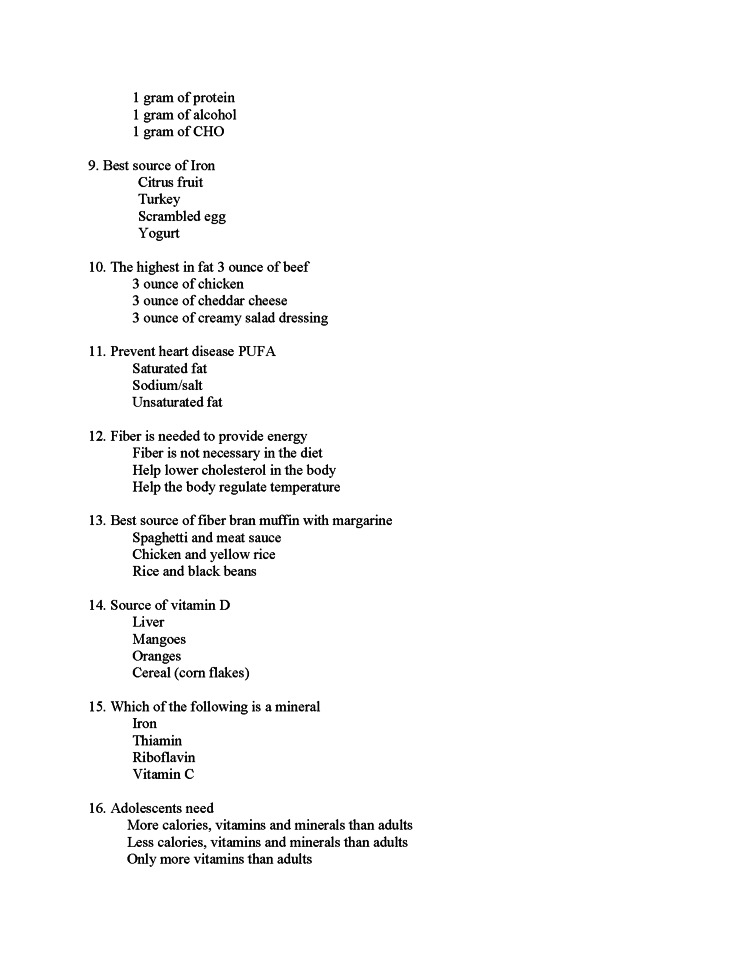
Comprehensive questionnaire (Contd.)

**Figure 10:**
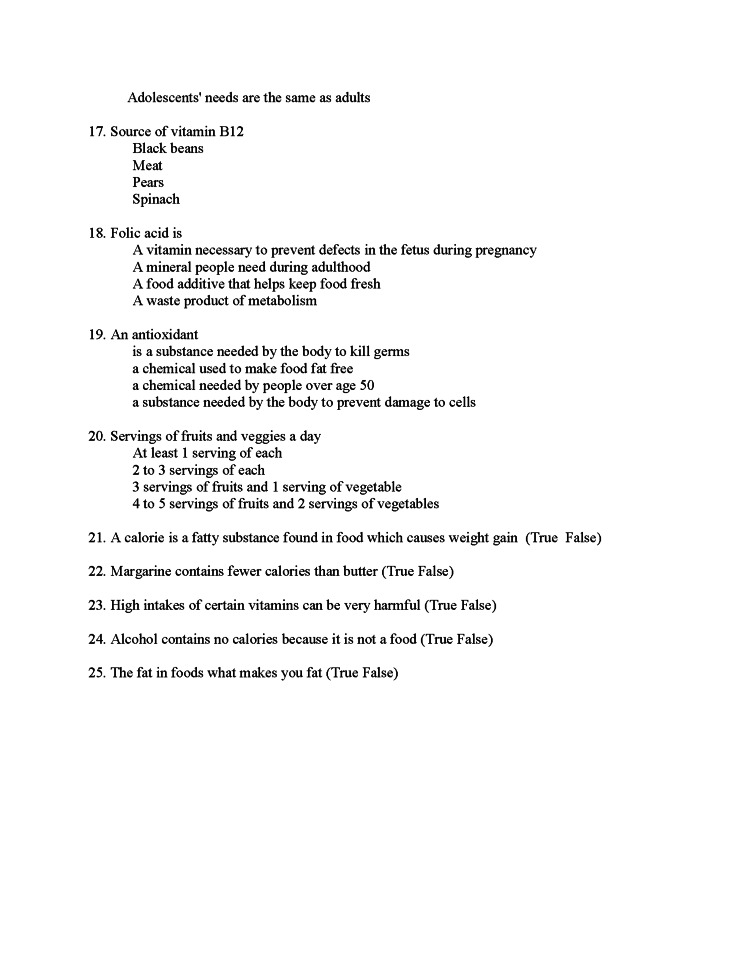
Comprehensive questionnaire (Contd.)

**Figure 11:**
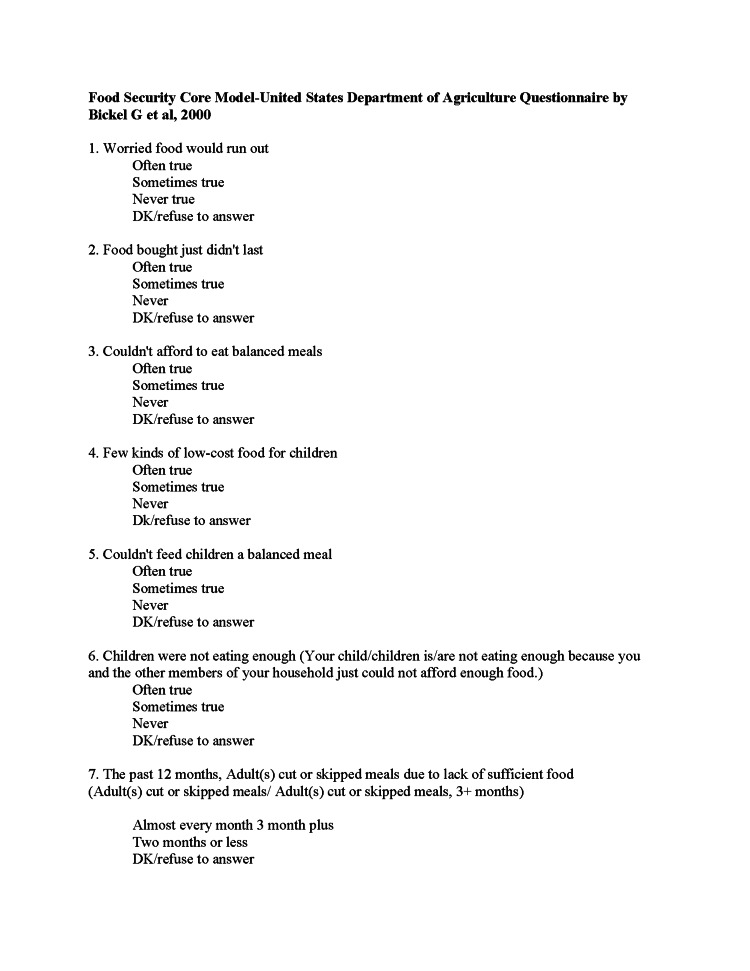
FSM-USDA questionnaire FSM-USDA: Food Security Core Model-United States Department of Agriculture

**Figure 12:**
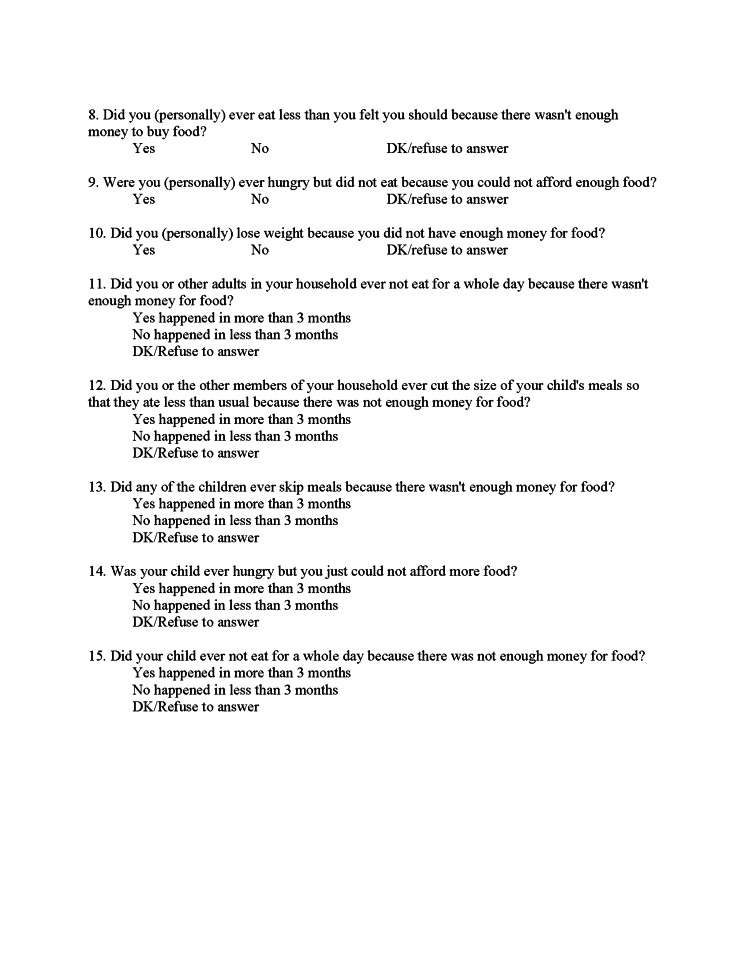
FSM-USDA questionnaire (Contd.) FSM-USDA: Food Security Core Model-United States Department of Agriculture

## Appendices

Appendix A

Appendix B
